# The Demobilised Doctor

**Published:** 1919-03-08

**Authors:** 


### The Hospital

The Workers' Newspaper of Administrative Medicine and Institutional Life, Administration, National Insurance and Health.

No. 1709, Vol. LXV. SATURDAY, MARCH 8, 1919. PRICE TWOPENCE

### THE DEMOBILISED DOCTOR.

Judging from present pronouncements of those responsible for medical demobilisation there will soon be an increased percentage of medical men released from service, and thereby the outcry for more care of the civil population answered. But the present tendency to consider primarily figures relating to the insurance panels suggests that the return and development of the future specialised worker, be he surgeon, clinician, or laboratory trained, has in many quarters been forgotten. Shortly after the armistice was signed, medical men were released in two main groups—the general practitioners urgently needed in particularly depleted areas, and the previously established specialists applied for personally by the hospitals to whose staffs they belonged. Such action made for the re-establishment of pre-war conditions as far as the exigencies of war allowed, but was at best a preliminary step and made no provision for the future. Later, when release was guided more and more by length of a man's war service, the situation became rapidly modified. New men, as they qualified, were called to the Colours to take the place of those whose war duties were already done, and thus, at the present moment, there is in the country a large number of that generation of doctors who joined up early and broke off their training, both academic and practical, at various stages of completion. They are now ready and anxious to take part in the profession's national work. Among them should be found many great men of the future who, had there been no war, would already have made their names known for other than Service activities. The fact that they are now in great need of opportunity both to gain further hospital experience and to find an outlet to bring their efforts to usefulness is obvious to any one who takes note of the number of such men now applying and advertising for appointments.

One hears that, in these times, the majority of young men wish to avoid general practice, and hence their difficulties; but it must be remembered that with every increase of medical knowledge—and many indeed the war has brought—there becomes more and more urgency that some men should have a thorough knowledge of one branch of medicine rather than an average facility in many. Modern problems of disease are to be elucidated by united rather than solitary effort. Team work is the key-note of the day. The principle of the coming Ministry of Health is understood to be actuated by that idea, and these men are justified in wishing to train for a place in the team. To a large extent it is the war that has produced the opportunity for reform, and the present returning groups of men have by their early response to its call well earned the opportunities they desire. That this is realised by the majority of governing bodies and institutions, and that the principle enunciated by the Central Medical War Committee on February 5, 1919, will be followed by most of them, there is no doubt. But there have been and still are many instances of delay in the practical application of these suggestions. The Committee, in its resolution, adheres to the principle that, where it is by any means possible, vacant appointments in institutions, etc., should be given to medical officers who have served with the Forces; that exceptional circumstances should be the only reason for departure from this rule; and that where it is not possible to appoint a demobilised officer a temporary appointment only should be made pending general demobilisation. If this programme is carried out in the correct spirit, it is certain that not only would justice be done, but also that more use will be made of that medical generation, the development of whose potentially brilliant members may be otherwise entirely lost to the nation.

